# Dimensioning of Wide-Area Alternate Wetting and Drying (AWD) System for IoT-Based Automation

**DOI:** 10.3390/s21186040

**Published:** 2021-09-09

**Authors:** Mushran Siddiqui, Farhana Akther, Gazi M. E. Rahman, Mohammad Mamun Elahi, Raqibul Mostafa, Khan A. Wahid

**Affiliations:** 1 Department of Electrical and Electronic Engineering, United International University, United City, Badda, Dhaka 1212, Bangladesh; mushran@live.com (M.S.); liza.farhana15@gmail.com (F.A.); rmostafa@eee.uiu.ac.bd (R.M.); 2 Department of Electrical and Computer Engineering, University of Saskatchewan, Saskatoon, SK S7N 5A9, Canada; khan.wahid@usask.ca; 3 Department of Computer Science and Engineering, United International University, United City, Badda, Dhaka 1212, Bangladesh; mmelahi@cse.uiu.ac.bd

**Keywords:** AWD, IoT, WSN, smart irrigation

## Abstract

Water, one of the most valuable resources, is underutilized in irrigated rice production. The yield of rice, a staple food across the world, is highly dependent on having proper irrigation systems. Alternate wetting and drying (AWD) is an effective irrigation method mainly used for irrigated rice production. However, unattended, manual, small-scale, and discrete implementations cannot achieve the maximum benefit of AWD. Automation of large-scale (over 1000 acres) implementation of AWD can be carried out using wide-area wireless sensor network (WSN). An automated AWD system requires three different WSNs: one for water level and environmental monitoring, one for monitoring of the irrigation system, and another for controlling the irrigation system. Integration of these three different WSNs requires proper dimensioning of the AWD edge elements (sensor and actuator nodes) to reduce the deployment cost and make it scalable. Besides field-level monitoring, the integration of external control parameters, such as real-time weather forecasts, plant physiological data, and input from farmers, can further enhance the performance of the automated AWD system. Internet of Things (IoT) can be used to interface the WSNs with external data sources. This research focuses on the dimensioning of the AWD system for the multilayer WSN integration and the required algorithms for the closed loop control of the irrigation system using IoT. Implementation of the AWD for 25,000 acres is shown as a possible use case. Plastic pipes are proposed as the means to transport and control proper distribution of water in the field, which significantly helps to reduce conveyance loss. This system utilizes 250 pumps, grouped into 10 clusters, to ensure equal water distribution amongst the users (field owners) in the wide area. The proposed automation algorithm handles the complexity of maintaining proper water pressure throughout the pipe network, scheduling the pump, and controlling the water outlets. Mathematical models are presented for proper dimensioning of the AWD. A low-power and long-range sensor node is developed due to the lack of cellular data coverage in rural areas, and its functionality is tested using an IoT platform for small-scale field trials.

## 1. Introduction

Rice (Oryza sativa) is the second highest ranked food staple in the world, feeding one third (2.6 billion) of the population (7.8 billion). About 90% [1] of the world’s rice is produced in Asia, and it is the fastest growing staple food in Africa and Latin America [2] due to its ease of preparation compared to that of corn and wheat. It provides 21% of global human energy per capita and is the largest single source of energy [3]. A total of 75% of world rice is produced in 92 million hectares of irrigated lowland, and Asia occupies 56% of this land [4]. Irrigated rice uses up to 43% of the world’s irrigated water, which is two to three times more than other irrigated crops, and thus places an excessive demand on water usage compared to other staple crops. 

Bangladesh, an Asian country with a growing population and an increasing demand for rice, is playing an important role in rice research. According to the Bangladesh Rice Research Institute (BRRI), the main source of irrigation for rice is from ground water. However, the ground water is retrieved using shallow tube wells (STW) and deep tube wells (DTW), which is resulting in deteriorating ground water levels [5]. The production of 1 kg of rice requires 3000–5000 L of water [6], and Boro rice, which is generally grown in dry months, predominantly relies upon the groundwater. In order to address this situation, the International Rice Research Institute (IRRI) developed an irrigation method called alternate wetting and drying (AWD) that requires 30% less water than the traditional irrigation method used during the growing phase [7]. Studies presented in [8] have shown that, compared to the traditional method, AWD requires five fewer irrigation cycles and fewer pumps which saves about 40% of fuel. Besides minimizing the use of groundwater, AWD reduces irrigation cycles without affecting the yield [9]. Due to intermittent flooding in AWD, emission of the greenhouse gas methane (CH_4_) is reduced by 73% in the dry season and by 21% in the wet season [10], compared to the continuous flooding method. This also decreases arsenic concentration in the shoots of rice plants [11].

Unlike other countries adopting AWD, where water is charged based on volumetric usage, most of Bangladesh’s irrigation water is charged per hectare, without any incentives for the farmers to reduce their water usage. Sharing a single pump amongst the farmers often gives rise to conflicts that usually deter any collaborative effort [11], which is a key point of AWD. The lack of proper knowledge and information sharing are also limiting the adoption of the AWD method. Research has shown that there is a significant opportunity for AWD in Southeast Asian countries such as Bangladesh and Vietnam [12]; however, due to social, economic, and technical challenges, the AWD method is not well perceived in these regions [13]. 

**Figure 1:**
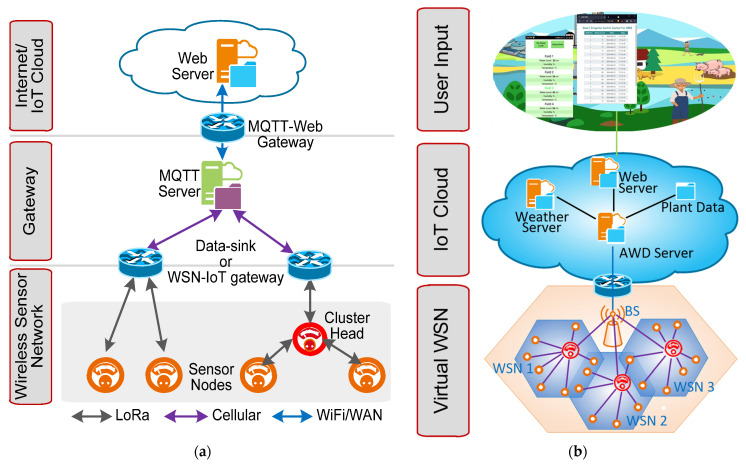
(**a**) Network elements and functional layers of the IoT-connected wireless sensor network (WSN), and (**b**) Automated AWD-specific WSN with IoT connectivity.

Besides the challenges of AWD described above, applying AWD to a wide area poses additional challenges, such as monitoring and controlling the complex mechanism, before it can become economically feasible. To achieve this, an automated IoT-connected wireless sensor network (WSN) is required. WSN is a network of sensors that accumulates monitoring data in centrally located storage, such as cloud storage. Most WSNs require three different types of network elements including the sensor node (SN), the cluster head (CH) and the data sink (DS), also called a base station, as shown in Figure 1a. All of the SNs are distributed into multiple, unequal clusters headed by a CH that sends sensor data to the DS. Functionally, the clustering process and data transfer protocol may vary depending on the nature of the application and the distribution of the SNs. Recent applications also require these WSNs to be connected with other networks through gateways and to the internet infrastructure [14], as shown in Figure 1a.

IoT is the network of various smart devices to facilitate information flow to the end-user through the existing internet. The exponential growth and distribution of IoT devices are giving rise to numerous innovative and real-life applications. COVID-SAFE [15] monitors and predicts the infection by monitoring social distancing with the help of wearable IoT devices. Various wearable IoT devices are used for human emotion recognition [16] to facilitate online communication. The growth of the IoT applications, such as smart health using wearable IoT devices and smart cities, results in security threats to the network itself. The authors [17] proposed an IoT Botnet Detection using salp swarm algorithm (SSA) and ant lion optimization (ALO) models to minimize security threats. Authors of [18] proposed an enhanced information-centric networking (ICN) for IoT using artificial intelligence (AI) and edge computing in the internet cloud. Studies are conducted on the design and fabrication technologies to utilize smart IoT devices [19]. The researchers focus on IoT nodes’ accurate and fast localization [20].

IoT connected to WSN has been implemented on a limited scale in agriculture as reported in [21,22,23,24]. Most WSN- and IoT-based irrigation automation systems utilize soil moisture content and other environmental variables to schedule irrigation without considering plant growth; however, this is insufficient for automating the AWD method and gaining the farmers’ trust. Besides monitoring and controlling the pumps, an effective water distribution system through pipes is also required, giving rise to the added complexity of having to both monitoring and controlling the distribution system. Moreover, present WSN technologies are optimized for applications with densely populated SN and small area coverage. Wide-area monitoring requires long-range and low-power wireless connectivity [25]. Therefore, wide-area AWD automation involves three different WSNs, dedicated for (1) field water-level and environmental monitoring; (2) irrigation system monitoring; and (3) irrigation system controlling, as shown in Figure 1b. Therefore, densely populated and widely distributed sensor and actuator nodes are required for these three different WSNs. These three WSNs will be interconnected virtually. Traditional WSN virtualization involves multilayer connectivity and control, which increases inter-layer WSN traffic [26] for the AWD application. Besides wide-area coverage, automated AWD also requires external parameters, such as real-time weather forecast data, plant physiological data, and the farmers’ input. These data can be used by connecting the WSNs with different servers throughout the IoT network, as shown in Figure 1b.

Literature shows that large-scale irrigation is required to limit water usage to provide more water for the growing industrial sectors and increasing urban population [27,28]. Large-scale multiple cropping can further limit water usage [29] using the automated irrigation framework proposed in this article. Despite the inherent advantages of the AWD, both small- and large-scale implementations are yet to achieve the expected success for lack of proper adaptation of technologies, namely IoT and WSN [30]. Moreover, the large-scale implementation of an irrigation system, such as AWD, involves multiple stakeholders and different technologies. That may require an effective dimensioning tool for a practical convergent solution. 

The research presented in this article has focused on automation through proper technology adoption by providing a mathematical model and addressing all the required systems. The proposed mathematical model considers the irrigation system as a distributed system. It separates its components into pipe networks, localized water control, and clustered pumps with centralized control, thus making the automation platform applicable to AWD and non-AWD irrigation systems. The pipe network will facilitate both micro- and macro-level irrigation control in a scalable manner. The distributed health monitoring of the pipe network and the clustered pumps will make the system more fault-tolerant. Various field-level sensors in an IoT-connected WSN will help build a long-term database enabling scientific research to study the impact of climate change on crop yield [31], physiological changes, greenhouse gas emissions for different crops, and the application of various irrigation models.

The major contributions of this paper can be summarized as follows.

(1) Develop a dimensioning model for large-scale implementation of AWD using pipes, actuator-based water distribution, and clustered pumps.
(2) Design WSN architecture combining three different functionalities with IoT-based automation for scalability and effective control and operation of the overall system.
(3) Develop a low-power and long-range (LoRa) sensor node and data sink using LoRa, 3G, and WiFi interfaces for field monitoring using an IoT network.
(4) Sample automation algorithms for the closed-loop AWD control using various internal and external parameters that can be used in both the WSNs and the IoT connected network.
(5) Provision for scientific research through the database of long-term sensor data and applying Machine Learning (ML) for future wide-area implementation.

The rest of the paper is organized as follows. Section 2 reviews existing literature related to the automation of AWD. Section 3 discusses the problem formulation for the automation of large scale AWD while Section 4 provides the proposed solution. Section 5 describes the system design and integration of WSN with AWD. Section 6 provides a description of the related field trial and Section 7 describes the performance of the system. Section 8 concludes the paper outlining future plans and work needed on this topic.

## 2. Related Work

Irrigation automation is not a very recent phenomenon and, as a result, is usually performed by adopting existing mathematical models and optimization algorithms. These primarily control the irrigation pumps to maximize water utilization. Baki et al. [32] have proposed a numerical scheme that utilizes weather forecasts and the water flow in soils to determine the optimal irrigation depth on scheduled irrigation days for the highest irrigation efficiency and optimum crop yield. Shahdany et al. [33] developed a mathematical model to automate canal irrigation using both known and unknown water demands for different irrigation systems. Besides the model-based optimization approaches, machine learning is also used by [34] to predict crop irrigation requirements based on soil parameters. Linker et al. [35] proposed a real-time and sub-optimal irrigation scheduling system that utilizes weather data alongside the crop’s physiological state and soil water availability. However, users need to decide between yield and irrigation performance depending on their applications. To achieve better optimization, Munir et al. [36] included parameters such as the plant type, watering time, air humidity, light intensity, and soil type for their fuzzy logic-based irrigation scheduling system that uses low power sensor-nodes. In [37], the authors designed an irrigation controller prototype based on the fuzzy rule to inspect water wastage by providing an optimal irrigation environment for farming. An artificial neural network (ANN) is used by [38] to predict soil moisture changes and to schedule irrigation based on climatic information and rooting depth. This ANN method was compared with the established water stress (WS) method, which well predicted the soil moisture during the main growing season, but was error prone at lower soil moisture levels. ANN and non-linear regression (NLR) were used by [39] to find the distances between the emitters in order to achieve optimum drip irrigation in the wet areas. Human expertise is also used by [40] for their proposed online decision support system (DSS), which consists of a meteorological model for weather forecasts, and a computational model for soil water balance and irrigation scheduling. It generates an irrigation schedule for a week in advance based on the DSS data repository.

Irrigation automation is further upgraded by utilizing various sensors, wireless links, and internet infrastructure due to the increasing availability and decreasing cost of these technologies. Water level-based scheduling was proposed by [41] and uses a low-cost water level sensor along with inputs from the local farmers through the IoT. In [42], the authors present a framework using an IoT-based water distribution and management system that utilizes water flow meters, ultrasonic sensors and motors. The over-usage of freshwater for flood irrigation is addressed by [43]. The author proposed an IoT-based intelligent decision support system to improve the farmer’s water utilization by using weather data and sensor data. A complete irrigation supervisory control and data acquisition ( SCADA) with WSN was implemented by [44] to compare seven different irrigation scheduling algorithms and found that soil-based approaches were not adequate in providing sufficient water for the plants.

Recently, more irrigation research on automated AWD systems is being reported in the academic literature. Localized pump automation is achieved by sensing the water level lacking remote monitoring or IoT connectivity and using solar panels as the power source [45]. An IoT-based system integrates laser sensors for precise water level measurement for automation of the AWD technique [46]. The proposed system was implemented for different sizes of fields, from 0.7 acres to 55.8 acres.

Besides these local and remote irrigation automations, performance optimization of the WSN platform is also focused on by [47]. They developed a 3D ray-launching (3D-RL) deterministic algorithm to characterize the radio channel of LoRa and LoRa wide area network (LoRaWAN) in order to provide better connectivity to transmit sensor data in obstacle filled scenarios for irrigation automation. Cardoso et al. [48] have proposed a narrow band IoT (NB-IoT) and LoRa-based system to monitor and schedule irrigation using machine learning. 

Unlike irrigation automation, AWD requires additional parameters such as plant physiological data in order to automate the irrigation system. As discussed in the previous paragraph, some irrigation automation utilizes weather data or plant physiological information from the farmer; however, this platform targeted only a single pump as the irrigation control unit. Most of these solutions are offline, of the open-loop type, and do not synthesize the data from all of the different sources. They lack wide-area considerations and do not focus on the complexity that comes with the integration of multiple physical and logical WSNs with the IoT. Proper dimensioning of pipe-based water distribution networks, pump clustering, irrigation depth, and plant lifecycle-specific schedule mapping is absent. Therefore, any one of them individually or in combination may not be suitable for the automation of the AWD method in wide area applications, which is highly required for the socio-economic success of the AWD implementation.

## 3. Problem Formulation

**Figure 2:**
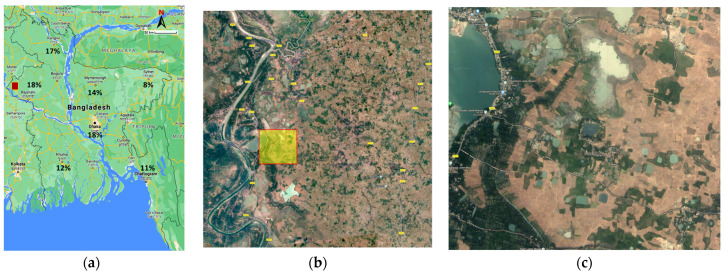
Boro production and illustrative implementation of AWD in Bangladesh; (**a**) Total Boro cultivation (2015–2016), (**b**) Portion of the medium Boro production zone focused for AWD implementation as marked in (**a**) by the red box, and (**c**) closer view of an IoT-based AWD system.

As one of the rice-producing countries of southwest Asia, Bangladesh produces three types of rice: Aus, Aman, and Boro. The north-west part of Bangladesh produces more than one-third of these three types of rice and 35% of Boro rice is produced in the country. Hence, the irrigated lowland of this part can be considered for the AWD implementation, as shown in Figure 2. Figure 2a shows the annual Boro production areas in overall Bangladesh.

For implementation of the wide area AWD, a portion of the medium Boro production area that produces less than 16% Boro [49] and lacks adequate irrigation facilities, as marked by the yellow box in Figure 2b which was chosen as the test site. Figure 2c shows the land condition of this area. The AWD zone is 5 km by 20 km, covering more than 24 thousand acres of cultivable lands that suffers from lack of irrigation. The closest water source is a river which is at least 2 km away from the cultivable land. Therefore, traditional canal-based irrigation methods are inadequate as they can suffer from water seepage of about 40% [50]. Furthermore, manual AWD is not able to provide efficient water level control over this large area of irrigated land. The proposed IoT-based automation system with pipe-based water transportation and distribution using the AWD system may reduce the seepage loss by up to 90% [51]. Local or centralized automation is not adequate in monitoring and controlling the large number of elements in the AWD system. Therefore, an intelligent layered IoT-based closed-loop automation system is required and is proposed for this project.

## 4. Proposed Solution

**Figure 3:**
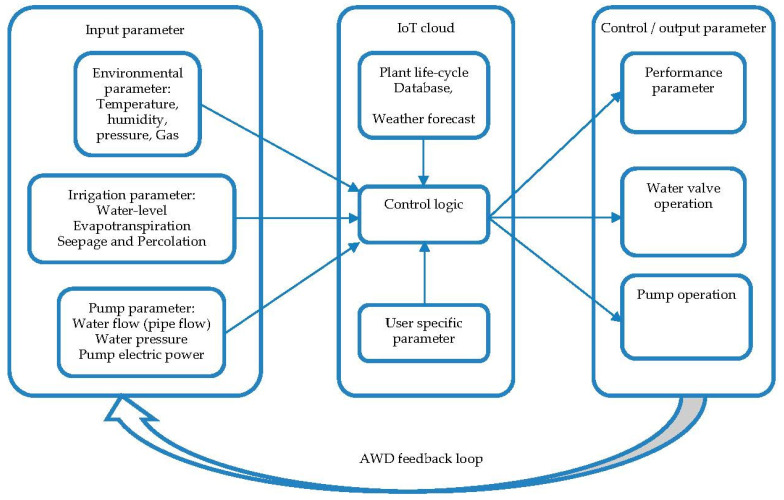
Functional blocks of the IoT–WSN-based AWD automation control loop.

The proposed automation of AWD is a closed-loop control system that uses an IoT based WSN, which is represented by a simplified block diagram as shown in Figure 3. This system has three different types of inputs, which are: (i) environmental inputs from the field and weather forecast server; (ii) irrigation inputs from a plant physiology database; and (iii) inputs from the users of the pumping network and water distribution system. 

The environmental parameters to be monitored are temperature, pressure, humidity, and the greenhouse gas methane ($CH_{4})$. Rice fields emit a considerable amount of methane, which can be reduced significantly by the intermittent flooding that occurs in the AWD method. The field temperature, pressure, and humidity data are required to calculate the evapotranspiration loss, seepage loss, and water level of the field, which are used to calculate the basic parameters used in the AWD method. Other environmental parameters, such as wind speed and daylight, can be collected from the weather forecast server through the IoT cloud.

The wide area AWD requires an adequate number of pumping machines and a pipe-based water distribution system to meet the field irrigation requirement. To maintain uniform irrigation throughout the area, the water pressures of the pipes need to be monitored continuously. The actuators for the water outlets and pumps are controlled to maintain proper pressure and water flow during the wetting phase of the AWD method. Additionally, the pump power and mechanical parameters need to be monitored continually. 

The crop coefficient ($K_{c\;}$) determines the AWD stages and can be determined from plant physiological data stored in the database and from input entered by farmers. The external data obtained from sensors in the field, the weather forecast, the crop coefficient, and user input are combined and processed in the closed control loop by the cloud-based AWD server through the IoT network. The control logic generated by the cloud server is then sent to the actuators and pumps through the IoT network to control the irrigation processes. All of the sensor nodes (SN) and actuator nodes (AN) are grouped into clusters and connected with the base-station (BS) through the cluster head (CH) using a LoRa wireless interface. The BS connects the whole WSN to the IoT network through the general packet radio service (GPRS) or third generation (3G) cellular network as per availability and coverage. The BS also acts as the WSN to an IoT gateway that converts the SN/AN data packets to message query telemetry transport (MQTT) messages. 

## 5. System Design and Integration

The proposed AWD automation consists of two main parts: (a) design and dimensioning of the AWD system; and (b) design and integration of the WSN. The AWD method at its core is an irrigation schedule that depends on certain the water requirements which vary depending on soil condition and the growth stages of the crop. Automation of the AWD using the IoT and WSN requires the sensor and actuator networks to be designed together, integration of various service modules, and development of the software algorithm. These are described in the following sub-sections. 

### 5.1. Irrigation Scheduling

The water volume of the irrigated field changes due to evapotranspiration and percolation. Evapotranspiration is influenced by the crop’s growing stage, wind speed, solar radiation, temperature, humidity, and soil properties. The crop evapotranspiration $\left(ET_{c}\right)$ [52] can be calculated as

(1) $$ET_{c}=ET_{0}\times K_{c\;}$$

where $\left(K_{c}\right)$ is the crop coefficient (potency of soil evaporation and crop transpiration), and $\left(ET_{0}\right)$ is the reference evapotranspiration. Among the different methods, the Food and Agriculture Organization’s (FAO) Penman–Monteith method [52] is considered to be the best estimation for $ET_{0}$, and can be expressed as

(2) $$ET_{0}=\frac{0.408\Delta \;\left(R_{n}-G\right)+\gamma \frac{900}{T+273}u_{2}\left(e_{s}-e_{a}\right)}{\Delta +\gamma \left(1+0.34u_{2}\right)}\;$$

where, $R_{n}=$ net radiation at the crop surface (${\mathrm{MJ}\;m}^{-2}d^{-1})$, *G* = soil heat flux density (${\mathrm{MJ}\;m}^{-2}d^{-1})$, T = mean air temperature (°C), Δ = slope of saturation vapor curve (kPa°C^−1^), $u_{2}$ = wind speed (${\mathrm{ms}}^{-1}$), $e_{s}$ = saturation vapor pressure (kPa), and $e_{a}$ = actual vapor pressure (kPa).

Water requirements for rice (*WRR*) in cm/day is given in Equation (3), which measures the receding water level.

(3) $$WRR_{j}=\left(ET_{o}\times K_{c_{j}}\right)+PERC$$

where *PERC* is the percolation loss from water seeping into the ground through pores, and *j* is the growth-stage index (1 to 3).

**Figure 4:**
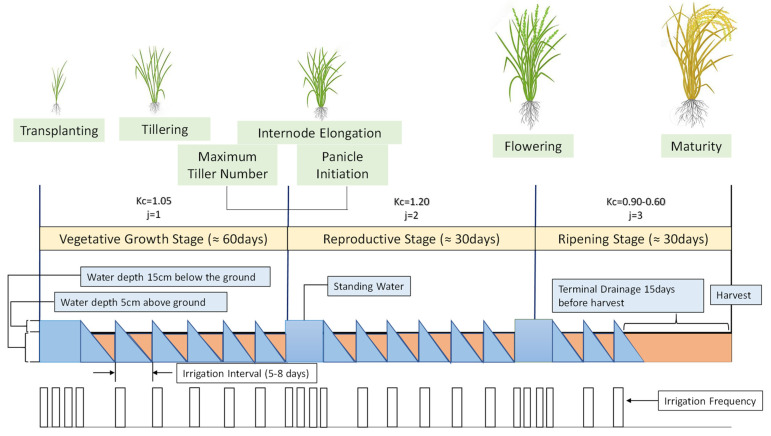
Irrigation scheduling in alternate wetting and drying (AWD) irrigation method.

Figure 4 illustrates the irrigation scheduling for the AWD method. The irrigation frequency (lower row) changes along with the development stages of the crop. Three development stages (transplantation, flowering, and panicle initiation) along with a water level indicator (middle row) are shown to indicate where continuous standing water and irrigation scheduling is required.

The different crop coefficients, including *Kc* of rice, for these three stages are shown in Figure 4 [53]. It is quite evident when AWD is initiated that, for approximately 5 to 8 days, depending on the soil type, no irrigation is required [54]. Continuous standing water is required at the beginning of all three stages which approximates to about 30 days in total; hence, utilization of the AWD method is suspended during these times. Therefore, depending on the stages and in accordance with the $WRR_{j}$, irrigation scheduling is applied. Irrigation scheduling is divided into two sections. The first section is for continuous standing water and the second section is for AWD period.

*Continuous Standing Water*:

For the three development stages, if the standing water level recedes to 2 cm from 5 cm as given in Equation (4), then the irrigation schedule or cycle $I_{1j}$ is applied using Equation (5). $I_{1j}$ represents the number of times the pump is turned on and is used to maintain a continuous water level of 5 cm above the ground. In this equation, TPF_j_ is the total number of days related to the transplantation, panicle initiation, and flowering stages.

(4) $$if\overset{T}{\underset{t=1}{\sum }}WRR_{j}\left(t\right)=3\;\mathrm{cm}\;in\;T\;days,\;j\in \left\{1,2,3\right\}\;$$

(5) $$I_{1j}=\frac{TPF_{j}}{T}$$

Establishment of continuous standing water during these stages is necessary as rice is quite sensitive to fluctuation in water level. 

*Alternate Wetting and Drying Method*:

For the AWD method, when the water level falls 5 cm from above the ground and reaches 15 cm below the surface as given in Equation (6), then irrigation $I_{2}$, which is the number of times the pump is turned on, is implemented using Equation (7), where $W_{j}$ is the total days for the AWD method, to bring the water level back to 5 cm above the surface.

(6) $$if\overset{T_{1}}{\underset{t=1}{\sum }}WRR_{j}\left(t\right)=5\;\mathrm{cm}\;in\;T_{1}\;days\;\;above\;the\;surface\;\;and\;\;Percolation\;loss\;=15\;cm\;in\;T_{2}\;days\;below\;the\;surface\;\;\;\;$$

(7) $$I_{2j}=\frac{W_{j}}{T_{1}+T_{2}}\;\;j\in \{1,2,3\}$$

The AWD method is utilized in three different stages of the plant development period: (i) 15 days after transplantation; (ii) then seven days before panicle initiation; and (iii) finally seven days after flowering.

### 5.2. Dimensioning of the AWD System

Proper dimensioning of the water distribution network’s pipes is required for the implementation of a large-scale AWD system. Multiple pumps are used in several clusters for a field of 25,000 acres to reflood the field in the shortest time possible. Multiple clustered-pumps facilitate the multiple inlet rice irrigation (MIRI) option available with AWD which allows the water to flow into the fields from several inlets, flooding the field in the shortest amount of time. The capacities of the pumps are chosen to meet the recommended minimum swift flooding rate of 0.00126 m^3^/s per acre (20 gallons per minute per acre), which varies depending on the type of soil [55]. Equation (8) shows the total number of pumps required (n), when operating for t hours with pumping capacity (c) in m^3^/s. The available pumping capacities include 0.02524, 0.05047, 0.06309, 0.1009, and 0.1262 m^3^/s, and are achieved with 400, 800, 1000, 1600, and 2000 GPM (gallons per minute) pumps that cover an acre of area (A) with *q* (150/m for one cm per hour water delivery) amount of water.

(8) $$n=\frac{A}{c\times q\times t}\;\;\;\;\;$$

**Figure 5:**
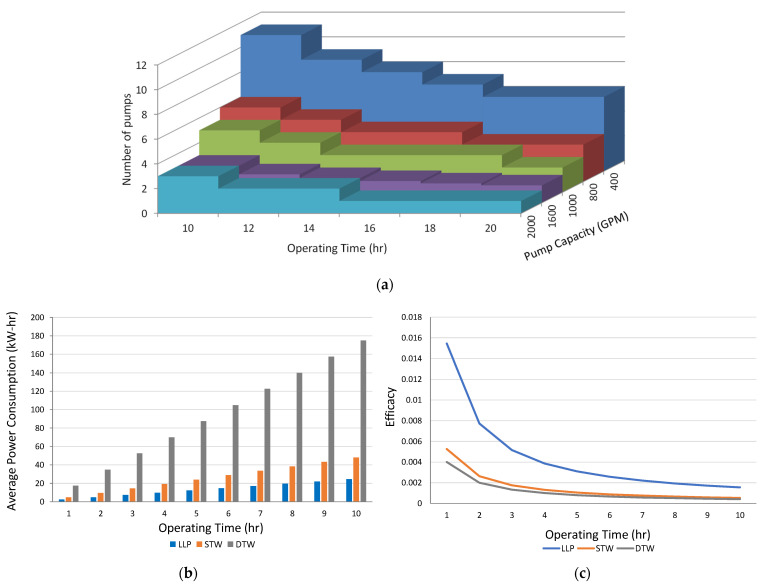
(**a**) Number of pumps required when operating for duration of time (**b**) Average power consumption between different water lifting devices (**c**) Efficacy of the water lifting devices.

Figure 5a shows the number of pumps required to irrigate 100 acre-inches. Using our proposed area of 25,000 acre-inches, it will require a total of 250 pumps with 2000 GPM capacities.

Beside the volume of water to be delivered, the selection of water pump depends on lifting head, water pressure, and parameters of the water sources. Depending on the lifting head and the source of the water, there are three different types of pumps used for irrigation in South-East Asian countries including Bangladesh. For the surface water on bodies of water such as rivers and lakes, low-lift pumps are used where the lifting head is between 1 to 5 m. A shallow tube well (STW) is used for more than 73% of ground water irrigation [56]. The rest is performed by pumps with higher lifting capacities called deep tube wells (DTWs). Irrespective of the type of pump used, pumps with higher efficiencies must be selected [57]. Pumps with 70% efficiency (as commonly used) are chosen for better selection among different lifting capacities. Average energy consumption (*Ea*) is calculated using Equation (9).

(9) Ea=hp·η· t 

where *hp* is the horsepower of the pumps, *η* is the pump efficiency, and *t* is the operating time of the pumps. The efficacy of the system is measured using Equation (10).

(10) $$efficacy=flow\;rate\;\left(m^{3}/s\right)/\;Ea\;$$

The average power consumption and efficacy of different water lifting devices are shown in Figure 5b,c. The efficacy curves in Figure 5c assume 600 GPM, 400 GPM, and 1110 GPM capacities for the low-lift pumps (LLP), STW, and DTW pumps. 

Figure 5b,c shows that LLPs have greater efficacy and low energy consumption compared to the other two types. This is mainly due to less heads [58] and thus makes the LLP type an economically feasible solution. Therefore, multiple low lift pumps are to be used for the wide-area AWD implementation as it would allow for proper utilization of the surface water and help lessen the exploitation of groundwater irrigation.

**Figure 6:**
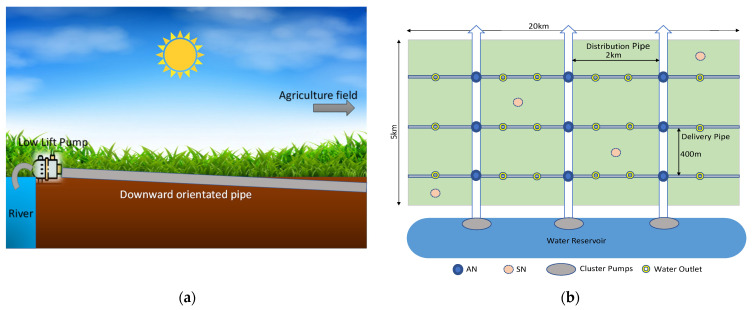
(**a**) Downward slope water pipe (**b**) In-field Automated AWD setup.

The proper dimensions of pipes are of paramount importance in carrying surface water to the field; therefore, pipes are chosen to ensure minimum friction loss while maintaining the recommended water flow velocity [59]. The pipes are placed on a downward slope, as shown in Figure 6a, to overcome the loss of pressure that occurs when travelling larger horizontal distances. Moreover, due to multiple inlets for irrigation, the pressure of water may fall from one inlet to the other; hence, when needed, the inlets closer to the source can be closed, to ensure sufficient water pressure at the opposite end of the field.

According to Equation (8), 250 units of 0.1262 m^3^/s (2000 GPM) capacity pumps need to be formed into clusters to ensure that the field is reflooded within 16 to 17 h of operating time. The frictional head loss for a steady pipe’s flow is calculated using the Hazen–Williams equation [59], which is given by Equation (11).

(11) $$h_{100ft}=\left(0.2083\times {\left(\frac{100}{c}\right)}^{1.852}\times q^{1.852}\right)/d_{h}{}^{4.8655}$$

where $h_{100ft}$ = friction head loss, in feet, of water per 100 feet of pipe, *c* = Hazen–Williams roughness constant, *q* = volume flow (gal/min) and $d_{h}$ = inside diameter of the pipe (inches). Subsequently, the friction loss in psi [59] can be calculated using the following Equation (12).

(12) $$f_{100ft}=\left(1/2.31\right)\times h_{100ft}$$

where, $f_{100ft}$ = friction loss in psi per 100 feet of pipe. Equations (11) and (12) are required to determine the diameter of pipes needed for specific applications. The holes of the inlet would need to be 0.0635 m in diameter to obtain a flow of 0.004732 m^3^/s (75 GPM) from every inlet in order to fill the field in the shortest possible time [55].

Table 1 shows the proposed AWD system with the number of monitoring and driver nodes needed to ensure that water gets dispersed evenly throughout the field. Figure 6b shows the AWD setup of the field, designed to ensure that all farmers receive a fair share of irrigation water, thus resulting in greater acceptance of the AWD.

### 5.3. WSN and IoT Integration

IoT-based automation of the AWD system requires different WSNs for sensors and actuators. All of these networks work independently with the same base station. A WSN collects field parameters such as water level, gas emissions ($CH_{4}$), temperature, pressure, and humidity. These data are then transmitted at 15 min intervals. The other WSN monitors the pipe and pump health parameters, such as pipe pressure, flow rate of water in pipes, and pump electric power. Based on these data, it generates alarms to shut down the pump in emergency situations. There is continuous monitoring at the SN which allows for immediate action when needed. The actuator network operates the actuators of the pipe to control the water flow through the pipe and is updated every 15 min.

Comparing the low-power wide-area network (LPWAN) technologies, LoRa was chosen for the WSN in order to achieve the wide area coverage needed at the monitoring area for the AWD automation. Table 2 summarizes the LPWAN technology features [14] including range, data rate, and power requirements. It shows that LoRa is the best suited technology in terms of range with higher data rates when compared to Sigfox.

LoRa employs chirp spread spectrum (CSS) modulation for better noise immunity and sensitivity in order to achieve low-power and long-range wireless connectivity. The LoRa data rate ($R_{b}$) depends on the channel bandwidth (BW), coding rate (CR), and the CSS spreading factor (SF), and can be calculated using Equation (13).

(13) $$\mathrm{Data}\;\mathrm{Rate}=\mathrm{SF}\frac{\left[\frac{4}{4+\mathrm{CR}}\right]}{\left[\frac{2^{\mathrm{SF}}}{\mathrm{BW}}\right]\;}$$

For the proposed automated-AWD system, the LoRa physical layer was chosen instead of LoRaWAN in order to customize the channel access interval and achieve better channel utilization by implementing a custom MAC (Medium Access Control) layer. Furthermore, WSN coverage can be improved by increasing the CSS spreading factor of LoRa, without increasing transmission power or changing any network elements [14]. Table 3 summarizes the LoRa configuration parameters used for the SN where the data rate Rb is calculated using Equation (13).

**Figure 7:**
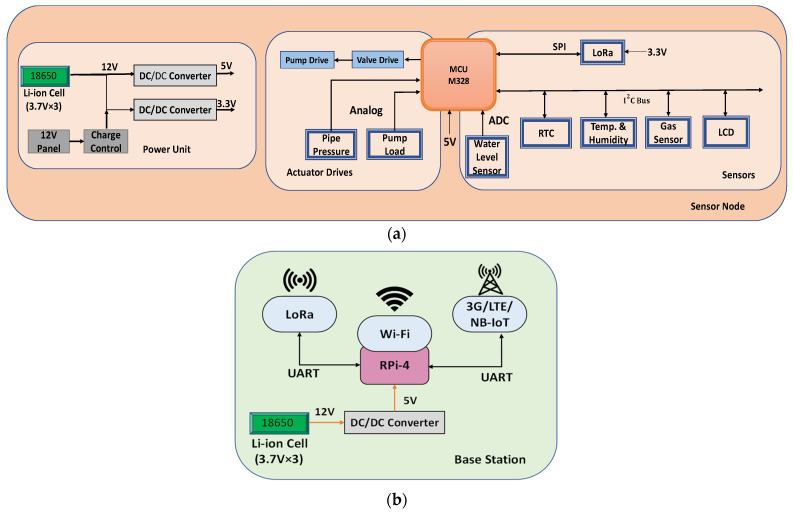
Hardware block diagram showing (**a**) Sensor node consisting of sensors, actuator drivers, and power unit; (**b**) base station (BS) with LoRa and cellular data connectivity.

The multi-purpose sensor node (SN), shown in Figure 7a, is designed for all WSNs in order to keep the network homogeneous. It consists of a LoRa transceiver module (SX1278) unit, main processor (ATMega 328), and network specific sensors and actuator drivers. Depending on the specific application, the sensor node can be used to either drive actuators or collect and monitor sensor data. Figure 7 shows the hardware blocks of the SN with the power unit and the base station.

The base station (BS) consists of a Raspberry Pi-4 which acts as a host computer to control the WSN, data communication, and gateway functionalities. It has both LoRa and Wi-Fi/3G/LTE to maintain the connection with the WSNs and IoT cloud. Figure 7b shows the hardware block diagram of the BS. 

### 5.4. Communication Protocol and Automation Algorithm

Data transmission from the SN is controlled by the BS. The BS initiates data request messages to a specific SN using their unique ID and sends an acknowledgement after successful reception of the sensor data. For the actuator node (AN), the BS sends the actuator control messages to the AN, and updates the actuator nodes in the server database after receiving the data and from the AN. All of these communications between the WSN and the BS are performed using LoRa with messages transferred as LoRa packets. The BS processes the LoRa data packets and sends them to the IoT server as MQTT messages. The commands used to control the pumps and actuator, sent from the IoT server, also use MQTT. On the user side, a smartphone app and web interface are connected with the IoT server through a cloud-implemented MQTT-hypertext transfer protocol (HTTP) gateway.

**Figure 8:**
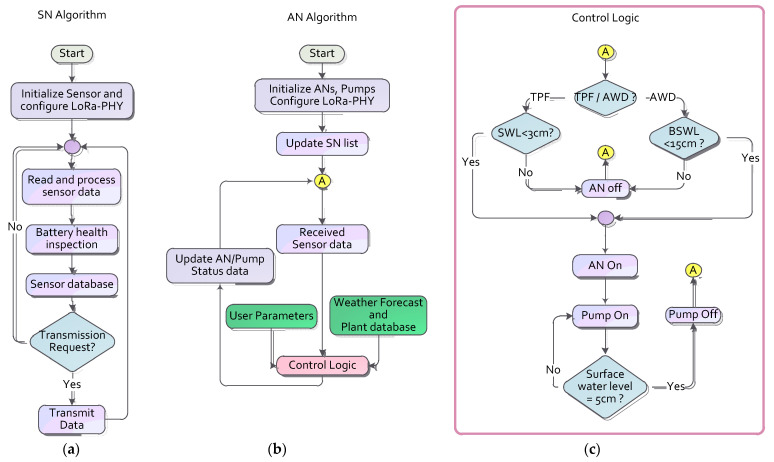
(**a**) Sensor node software algorithm used for field trial, (**b**) Proposed actuator node algorithm, and (**c**) Control logic algorithm for the AWD server intended for wide-area AWD automation.

All of the data communication and AWD automation processes are performed by the SN, BS, and cloud IoT server. Figure 8a,b show the possible software algorithms designed for the SN and AN, respectively. At first, it configures and initializes the sensors, LoRa module, and driver unit. It then starts reading the field, pipe, and pump sensors to monitor their condition continuously. The AN takes all necessary actions according to certain pump operating conditions and control logic defined by the user during the initialization phase. All the internal control variables are updated according to external parameters at regular intervals in a closed loop at node A. These external parameters, such as user input, plant-specific data, and weather forecasts, are collected through the IoT server. Figure 8c shows the AWD automation control logic performed by the IoT server. The SN algorithm, shown in Figure 8a, was used for the field trial (described in Section 6). The algorithms, shown in Figure 8b,c, intended for wide-area AWD automation, were not used in the field trial. Initially, the irrigation phase (TPF or AWD) is ascertained, at which point all of the phase specific decisions are taken. For example, the surface water level (SWL) is monitored in terms of TPF, and, for the AWD, the below surface water level (BSWL) is checked to determine whether to toggle the actuator nodes on or off. Subsequently, after toggling the actuators and pumps, the SWL is measured for both the TPF and AWD, and then, if the SWL reaches 5 cm above the surface, the actuators and pumps are switched off.

## 6. Field Trial

**Figure 9:**
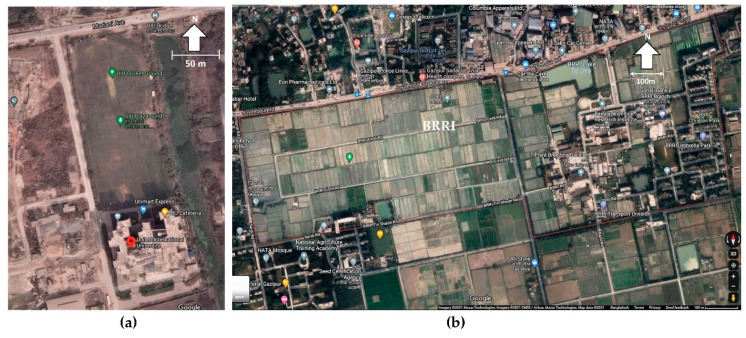
Field trial location: (**a**) On-campus trial at United International University. (**b**) Agricultural land trial at BRRI.

A field trial was performed in two phases: (i) WSN formation and data collection at UIU (United International University), Bangladesh; and (ii) remote data collection using the IoT network in an AWD plot at the Bangladesh Rice Research Institute (BRRI). Figure 9a shows the location of the UIU campus fields and Figure 9b shows the BRRI’s research agricultural field on Google Maps.

**Figure 10:**
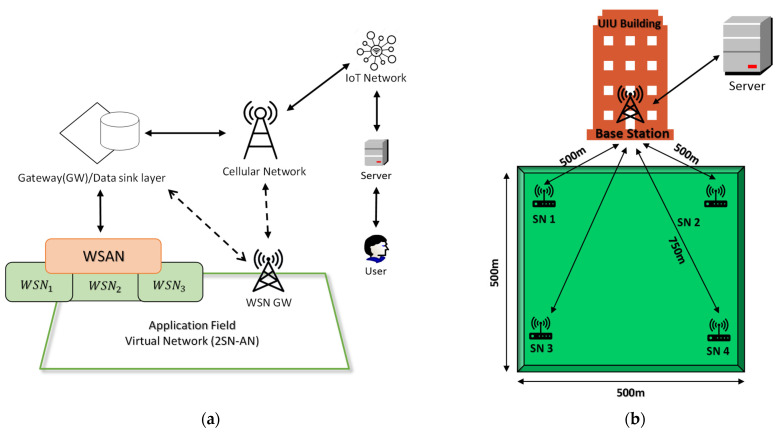
(**a**) IoT-connected WSAN architecture (**b**) On-campus trial setup with sensor devices connected to a base station IoT-connected WSAN architecture.

Figure 10a shows the IoT-connected (wireless sensor and actuator network (WSAN) architecture which is comprised of two sensor networks (WSN1 and WSN2) and one wireless actuator network (WSN3), as described in Section 5.3. In phase one, four sensor nodes (SNs) were placed at each corner of a 500 m by 500 m field with the indoor base station (BS) located in a lab on the fifth floor of the campus building. The approximate distances between the SNs and the BS were 500 m to 750 m, as shown in Figure 10b.

Sensor data were collected on a round-robin basis to avoid the co-channel interference that occurs at high frequencies (30 s period), and to evaluate the data transfer performance of the WSN. The base station (BS) consisted of a Linux-based (Redhat 7) Apache web server with Node.js, HTM, Python 3, and Maria Database. It also acted as a WSN-IoT gateway using the LoRa–WiFi interface.

**Figure 11:**
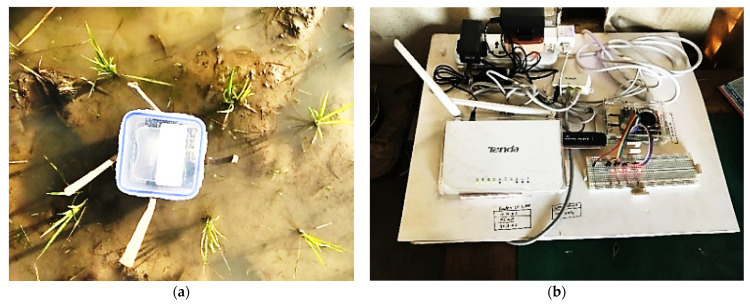
Agricultural field level trial setup: (**a**) Top view of a sensor device in the field (**b**) The receiver base station setup.

To collect real-life data and to evaluate the AWD algorithm’s performance, SNs were placed in the agricultural plots of BRRI in a Boro field. The field was divided into irrigation blocks of 15 m by 15 m, and the SNs were placed 200 m apart from each other. The irrigation system was controlled using a central pump. Figure 11 shows the field setup. 

**Figure 12:**
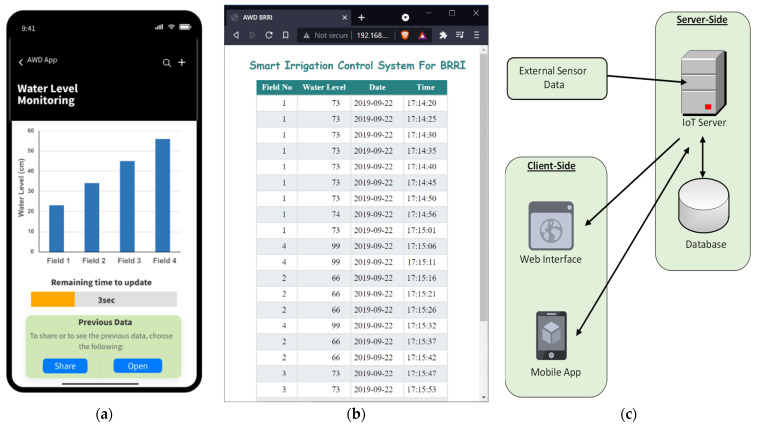
(**a**) Mobile Application, (**b**) Web Application, and (**c**) client-server connectivity for user interface.

The BS located at BRRI acquired all the sensor data from the field and stored it in the IoT server in a lab at UIU through the 3G cellular network. To monitor and process the field data in real-time, a distributed application structure was designed, as shown in Figure 12c. Here, a smartphone app, web interface, and other automation applications were developed and primarily implemented within the central IoT server [60]. Figure 12a,b show the smartphone app and the web interface. 

The mobile and web applications were linked to a Node.js server. A mobile Android application was used to collect data, a server was used to store the data and generate notifications, and a web client was used as a dashboard to display all the AWD specific information. The mobile app and web dashboard used rESTful APIs, HTTP requests, and web sockets to interface with the server. Sensor data from the external sensor devices was recorded on the server, where it was reactively pushed to the mobile client and dashboard using web sockets. Web sockets were used to communicate between the server and the clients. Both the Android and web apps provided similar services, such as user registration, sensor data acquisition, warning generation based on sensor data, and data and AWD status reports on the dashboard. The end user, farmers, can reconfigure the AWD parameters, such as water level and pump operation time using either the smartphone app or web interface.

## 7. Performance Analysis

In traditional irrigation systems, water distribution is performed using earthen canals [50] due to their low initial cost. However, these canals result in poor distribution efficiency, less area coverage, and, in the long run, results in high maintenance costs. The greatest concern in using earthen canals is the high water loss, which is expressed as water conveyance loss (*S*) [51] and can be calculated using Equation (14).

(14) $$S=\left\{\left(Q_{1}-Q_{2}\right)÷L\right\}\times 100$$

where *S* = the rate of conveyance loss in the canal ($m^{3}\;s^{-1}$) per 100 m distance, *Q*_1_ = rate of flow at the inlet ($m^{3}\;s^{-1}$), *Q*_2_ = rate of flow at the outlet ($m^{3}\;s^{-1}$), and *L* = distance between two points (m). 

**Figure 13:**
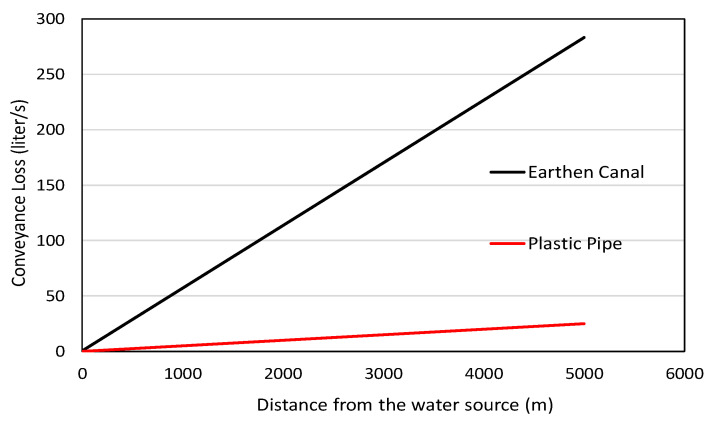
Conveyance loss compensation between earthen canal and plastic pipe.

The main factor causing this loss in the earthen canals is due to seepage loss. Studies have found that about 40% of the water is lost during the distribution time through the earthen canals [50]. The proposed automated AWD system using plastic pipes can overcome this conveyance loss, and help reduce the irrigation time, which in turn results in less energy (fuel or electricity) to operate the pumps. The conveyance loss rate for the proposed project derived from [51] is shown in Figure 13. It compares the conveyance loss between earthen canals and plastic piping when water is transported 5000 m from the water source to the field. Thus, the proposed design saves about 90.91% of water when transporting it from the source using plastic piping instead of earthen canals. Furthermore, studies conducted by [51] have found that the incorporation of the AWD method with plastic pipes has saved about 42% of water, and reduced energy consumption by 41.2% compared to the traditional method.

Using Equations (5) and (7), the frequency of irrigations, $I_{1j}\;\mathrm{and}$ $I_{2j}$, can be used to determine the total energy consumption of the pumps. For instance, to reflood a 100-acre field with 3 cm of water while maintaining continuous water during TPF_j_, a single 2000-GPM capacity pump with 70% efficiency and 12 hp, operating for 18 h, would be sufficient as seen in Figure 5a. Then, using Equation (9), average power consumption of the pump is found to be 142.8 KWh. Similarly, the average energy consumption can be determined for the AWD method. 

**Figure 14:**
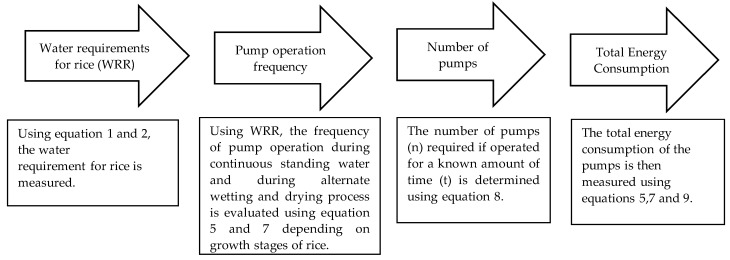
Required dimensioning and calculation steps for an IoT-based AWD system.

Therefore, the total energy consumption for the whole season can be found by using Equation (15):

(15) $$Total\;Energy\;Consumption\;\left(KWh\right)=I_{1j}*Ea\left(TPF_{j}\right)+\;I_{2j}*Ea\left(AWD\right)$$

where $Ea\left(TPF_{j}\right)$ represents the energy consumption during the TPF stages in order to supply 3 cm of water to the field, and Ea(AWD) denotes the energy required to supply water when water reaches 15 cm below ground during the AWD method. It can be observed that, compared to the traditional method which utilizes continuous water, the proposed automated system is expected to require less energy. Figure 14 shows the dimensioning steps and mathematical calculations that are used for the proposed AWD system.

Table 4 compares the automated AWD method with manual AWD systems. Manual AWD studies focused on limited field sizes, soil types, and water sources. Sharing a limited number of water-lifting devices amongst numerous farmers tended to cause conflicts among them. Furthermore, the number of farmers it supported was generally quite limited. However, in the proposed IoT- and WSN-based automated AWD system, large-scale implementation is possible using a pipe and actuator-based water distribution system with cluster pump operation and monitoring. In this proposed system, 10 clusters, each containing 25 pumps with capacities of 0.1262 $m^{3}$/s (2000 GPM), can cover an area of 25,000 acres. In this system, the pumps have 12 hp with an average pumping efficiency of 70%, which can support a large number of farmers while still allowing each farmer to obtain a fair share of water.

In manual AWD, due to manual inspection of water levels throughout the irrigation season, pumps may operate for more hours than necessary, resulting in the wastage of valuable resources. However, in automated systems, pumps are switched off as soon as the water level reaches a certain threshold level, resulting in water and energy savings. The experiments presented in [46] showed that the automated AWD saved between 13–20% of water, with a 25% decrease in irrigation energy cost, and an increase in rice yields by 2–11% over manual AWD. The AWD automation scheme proposed in this paper is expected to reduce irrigation costs further since it uses plastic pipes to carry water. Moreover, integration of weather forecast data will operate the pumps effectively (e.g., keeping pumps off when the forecast predicts precipitation).

Research in [45,46] presented automation of the AWD process by using sensors to precisely measure the water level precisely. These studies focused on small areas compared to that of the system described in this paper. For large-scale automation of AWD, multiple WSNs need to be designed and implemented. While automated AWD systems offer certain advantages over the manual AWD, as described in Table 4, the dimensioning of the wide-area automated AWD system may face challenges during implementation. The plant physiological database for developing countries such as Bangladesh is not regularly updated and is not easily accessible. In Bangladesh, where farmers are largely not exposed to technology, their knowledge and experiences about rice and irrigation are not readily available. Moreover, one of the research constraints is that the weather forecast information is not based on the specific local territory. Rather, it is based on the general meteorological information of the whole region.

## 8. Conclusions

This paper has presented an approach to automating a wide-area AWD system covering a total irrigation area of 25,000 acres. Implementation of AWD in such a large irrigation area has not been reported in the existing literature. It is imperative that such a large system uses automation instead of the traditional manual operation and monitoring approach. The automation can be achieved by integrating three essential and intricate wireless sensor networks (WSN): a mechanical subsystem comprised of pumps, actuator networks, and a water distribution network, and an IoT system that runs the automation algorithm and issues control commands. The data from the WSN, along with other operating parameters, are processed at a central server that decides whether to activate the pumps and the actuator valves. The system is scalable and the algorithm can be adjusted for irrigation areas with different sizes within a practical limit. The required set of equations to support the design and operation of the overall system is provided in this paper. The hardware for the system is presented and described in order to facilitate the implementation of the automation system. The communication protocol and automation algorithm have also been presented in sufficient detail. A sample study on a small-scale field trial is presented as a proof-of-concept to demonstrate the effectiveness of the proposed system. An analysis of the proposed system’s expected performance is discussed by comparing it with manual and automated AWD systems in the existing literature. The analysis highlights the large irrigation area and the complete automation aspect of the proposed system. 

Despite numerous advantages, such as reduced water consumption, and economic and environmental benefits, widespread adoption of AWD is yet to be materialized due to social, economic, and technical challenges. This paper demonstrates that, with the adoption of existing sensor networks and IoT-based technologies, wide area adoption [25] is feasible and economically viable. The proposed wide-area AWD system allows for continuous monitoring and real-time decision making along with a fair distribution of water amongst the farmers. The authors are optimistic that, with the adoption of the proposed system, wide area AWD will be implemented in rice-growing countries. These countries will then reap the full benefits of the technological advantages that AWD has to offer.

For future expansion of the proposed system, long-term data can be collected and analyzed to improve the system further and to further control the greenhouse emissions more effectively. Since the proposed system enables the collection of data and creation of a large database, new models can be generated and advanced techniques, including machine learning and big data, can be applied for better performance. Furthermore, incorporating the farmer’s valuable input alongside previous data and algorithms will make it possible to accurately identify the different physiological stages of plants during the season, resulting in the improved development of plants and yields.

## Figures and Tables

**Table 1:** Proposed AWD system setup.

Proposed Area (Acres)	Cluster of Pumps	Each Cluster Containing
25,000	10	25 pumps of capacity 0.1262 m^2^/s (2000 GPM)
**Pipes**	**Placements**	
Distribution Pipes	Every 2 km	
Delivery Pipes	Every 400 m	
**Nodes**	**Placements**	**Total Nodes**
Actuator Nodes	Every 400 m	400
Sensor Nodes	Every 1 km	100

**Table 2:** Basic features of wireless technologies.

Technology	Data Rate (bits/s)	Range (km)	Power Requirement
LoRa	<50 k	5 to 15+ (rural)	Very Low
Sigfox	600	10 to 50(rural)	Very Low
Zigbee	20 k, 40 k, 250 k	Less than 1	Very Low
Bluetooth	1 M	0.01–0.1	Low
Wi-Fi	Up to 54 M	0.2	High

**Table 3:** LoRa parameters.

Symbol	Description	Value
SF	Spreading Factor	7
CR	Code Rate	1
BW	Bandwidth	250 kHz
$R_{b}$	Data Rate	10.937 kbps
Tx Power	Transmission Power	+20 dBm
Rx Sensitivity	Receiver Sensitivity	−148 dBm

**Table 4:** Comparison between manual AWD and automated AWD.

AWD Parameters/Features	Manual AWD			Automated AWD		Proposed Automated AWD System
	[61]	[8]	[62]	[46]	[45]	
Research Focus	Case studies on five different locations regarding AWD applications	Comparison study between AWD and Traditional Method (TM)	Adoption rate of AWD in Bangladesh	Using IoT for precise water measurement to promote AWD irrigation in Vietnam	The usage of solar panels to power the sensors in-order to detect water level and to automate the AWD system	Dimensioning of the water distribution system with multiple pumps, valves and sensors for IoT-based automation of wide-area AWD.
Irrigation Area (acres)	6	5.58	16.4	0.7–55.8	The prototype was tested in 1 m^2^ field	25,000
Maximum Supported Users	9–40			Overall, 82 farmers at three different locations	Information Not available	1000+
Number of pumps supported/implemented	3–12 STW pumps			One local pump in the field	Not available	250 pumps in 10 clusters
Conveyance Loss Rate	Information not available, but expected to be high due to the use of earthen canals, for example, 6.1 L/s per 100 m [51].			Not available	Not available	Low due to the use of pipes. 0.5 L/s per 100 m (derived from [51])
Pump/irrigation control type	Manual pump control			Manual pump control	Automatic pump control	Automated pump and valve control
Accuracy of water level measurement	Depends on manual inspection			Depends on the single laser sensor	Depends on the single ultrasonic sensor.	Measured using multiple sensors in a wide-area WSN
Plant physiological data	Visually observed by the farmers			Information not available	Information not available	Previous data and farmers input can be used via IoT platform
Meteorological Information	Not used			Not available	Not available	Can be integrated via the IoT platform
Other advantages	Easier for farmers to learn this simple methodology			The usage of solar powered water level sensors	Fast charger requires less time to recharge the battery	Proper utilization of rain-water is possible.
Other issues	Prone to over usage of pumps, may cause flooding of the plot			Use of relatively costly sensors	Cannot work automatically without acceptable internet connection	Need multiple WSNs (Focused as our future research scope)

## Data Availability

Not applicable.
